# Genome-wide identification and bioinformatics analysis of the WD40 transcription factor family and candidate gene screening for anthocyanin biosynthesis in *Rhododendron simsii*

**DOI:** 10.1186/s12864-023-09604-x

**Published:** 2023-08-26

**Authors:** Cheng Wang, Yafang Tang, Yan Li, Chao Hu, Jingyi Li, Ang Lyu

**Affiliations:** 1 Key Laboratory for Quality Control of Characteristic Fruits and Vegetables of Hubei Province, College of Life Science and Technology, Hubei Engineering University, Xiaogan, 432000 China; 2 Department of Biology and Chemical Engineering, Weihai Vocational College, Weihai, 264200 China; 3 Institute of Quality Standard and Testing Technology for Agro-Products, Hubei Academy of Agricultural Science, Wuhan, 430064 China

**Keywords:** *Rhododendron simsii*, WD40, Duplication analysis, Anthocyanin biosynthesis, Candidate genes

## Abstract

WD40 transcription factors (TFs) constitute a large gene family in eukaryotes, playing diverse roles in cellular processes. However, their functions in the major ornamental plant, *Rhododendron simsii*, remain poorly understood. In this study, we identified 258 WD40 proteins in the *R. simsii* genome, which exhibited an uneven distribution across chromosomes. Based on domain compositions and phylogenetic analysis, we classified these 258 RsWD40 proteins into 42 subfamilies and 47 clusters. Comparative genomic analysis suggested that the expansion of the *WD40* gene family predates the divergence of green algae and higher plants, indicating an ancient origin. Furthermore, by analyzing the duplication patterns of *RsWD40* genes, we found that transposed duplication played a major role in their expansion. Notably, the majority of *RsWD40* gene duplication pairs underwent purifying selection during evolution. Synteny analysis identified significant orthologous gene pairs between *R. simsii* and *Arabidopsis thaliana*, *Oryza sativa*, *Vitis vinifera*, and *Malus domestica*. We also investigated potential candidate genes involved in anthocyanin biosynthesis during different flower development stages in *R. simsii* using RNA-seq data. Specifically, we identified 10 candidate genes during the bud stage and 7 candidate genes during the full bloom stage. GO enrichment analysis of these candidate genes revealed the potential involvement of the ubiquitination process in anthocyanin biosynthesis. Overall, our findings provide a valuable foundation for further investigation and functional analysis of *WD40* genes, as well as research on the molecular mechanisms underlying anthocyanin biosynthesis in *Rhododendron* species.

**Supplementary Information:**

The online version contains supplementary material available at 10.1186/s12864-023-09604-x.

## Introduction

The WD40 transcription factors (TFs), also known as WD-repeat (WDR) proteins, constitute one of the largest TF families in eukaryotic organisms [1]. The WD40 TF is distinguished by the existence of multiple WD40 repeats, each consisting of 44–60 amino acids. These repeats adopt a seven-bladed β-propeller structure formed through repetitive folding. The N-terminus of each blade contains a glycine-histidine (GH) dipeptide with 11 to 24 residues, while the C-terminus is composed of the WD dipeptide [2, 3]. This arrangement allows for the formation of a stable repeat fold with a robust hydrogen bond network [4]. Besides the WD40 repeats, WD40 proteins often contain additional protein domains, resulting in lower sequence conservation among family members [5]. Different researchers have classified WD40 TFs in various ways, either based on phylogenetic analysis or domain compositions. For instance, in *Arabidopsis thaliana*, 237 AtWD40 TFs with a minimum of 4 WD40 repeats were grouped into 33 clusters, while in cucumber (*Cucumis sativus*), 191 CsWD40 TFs were categorized into 21 clusters [6]. Phylogenetic analysis divided 167 GbWD40, 204 FcWD40, 743 TaWD40, 225 SiWD40 and 187 RcWD40 TFs from *Ginkgo biloba*, *Ficus carica*, wheat (*Triticum aestivum*), *Setaria italica*, and *Rosa chinensis*, respectively, into 5 clusters, while domain composition analysis categorized them into 16, 12, 11, 12, and 15 subfamilies [7–11]. In apple (*Malus domestica*), 346 MdWD40 TFs were classified into 14 clusters based on phylogenetic analysis and 12 subfamilies based on domain composition analysis [12]. Similarly, in Potato (*Solanum Tuberosum*), 178 StWD40 TFs were grouped into 14 clusters based on the phylogenetic tree and 10 subfamilies based on domain compositions [13]. Additionally, phylogenetic analysis of 315 MiWD40 TFs from mango (*Mangifera indica*) and 231 CmWD40 TFs from *Cucurbita maxima* resulted in their classification into 11 and 5 clusters, respectively [14, 15].

WD40 TFs are typically recognized as adaptors that recruit other transcription factors to form protein or protein-DNA complexes [16, 17]. They play versatile roles in various physiological and biochemical processes, including cell cycle and division [18–20], DNA damage repair [21, 22], signal transduction [23, 24], flower development and flowering [25–27], hormones responses [28, 29], and stress responses [30–32]. One prominent function of WD40 is its involvement in the regulation of anthocyanin biosynthesis through the MYB-bHLH-WD40 (MBW) complex. The *WD40* gene *TTG1* was initially identified in *Arabidopsis*, and mutations in this gene (*ttg1-1*) result in yellow seeds due to the absence of anthocyanin accumulation [33–35]. TTG1 can form MBW complexes with R2R3 MYB transcription factors PAP1, PAP2, MYB113, MYB114 or TT2, and bHLH transcription factors GL3, EGL3 or TT8 [35–41]. Different MBW complexes regulate the biosynthesis of different flavonoids, and it has been demonstrated that the TTG1-TT8/GL3-PAP1/PAP2/MYB113/MYB114 complexes activate the expression of late biosynthetic genes, such as dihydroflavonol-4-reductase (DFR), anthocyanidin synthase (ANS), and UDP-glucose: flavonoid-3-O-glucosyltransferase (UFGT) in the anthocyanin biosynthetic pathway [38, 42, 43]. The TTG1-GL3/TT8-TT2 complexes activate the expression of DFR, ANS, BANYULS (BAN), TT19, and TT12 to regulate anthocyanin accumulation [37, 40, 43]. While *TTG1* orthologs in various plant species, such as *Camellia sinensis *[44], *Salvia miltiorrhiza *[45], *Vitis vinifera *[46], rice [47], and *Dendrobium candidum *[48], have been shown to regulate biosynthesis and accumulation of anthocyanin, no other *WD40* genes have been identified in anthocyanin biosynthesis pathway. Consequently, there is a lack of literature investigating the role of *WD40* genes in flower coloration, particularly in plants with a diverse range of flower colors. Therefore, further research is necessary to explore the potential involvement of *WD40* genes in anthocyanin synthesis.

The *Rhododendron* genus (Ericaceae) is renowned for its extensive variety of corollas, encompassing more than 1000 species and 30,000 cultivars [49, 50]. *Rhododendron* exhibits a wide range of flower colors, including red, light red, pink, white, yellow, and blue. The aesthetic and economic value of *Rhododendron* as an ornamental plant is greatly influenced by the diversity of its flower colors. In *Rhododendron* species, flower pigmentation predominantly relies on the presence of anthocyanins and flavanols. Specifically, the composition and quantity of anthocyanin constituents determine the flower coloration [51, 52].

To date, there has been no comprehensive investigation of the *WD40* gene family in *R. simsii*. In this study, we performed a genome-wide analysis of *R. simsii WD40* (*RsWD40*) genes based on the published *R. simsii* genome [49]. We identified a total of 57 *RsWD40* genes. Subsequently, we examined their chromosome locations, categorized them into different groups, established their phylogenetic relationships, and investigated gene duplication events among the *RsWD40s*. Furthermore, we identified candidate *RsWD40* genes associated with anthocyanin biosynthesis using RNA-seq data from *Rhododendron* flower samples from three varieties with distinct flower colors. The findings of this research enhance our understanding of the *RsWD40* gene family and will facilitate genetic improvements in flower coloration in *Rhododendron* species.

## Materials and methods

### Investigation of WD40 TF family in *R. simsii* and other plant species

The complete protein sequence data of *R. simsii* was obtained from the Rhododendron Plant Genome Database (RPGD), accessible at http://bioinfor.kib.ac.cn/RPGD/. To identify potential RsWD40 proteins, we used the Hidden Markov Model (HMM) profile for WD40 (PF00400) as a query and performed a search using the HMMER 3.0 software [53]. The putative RsWD40 proteins were then validated by submitting them to the SMART [54] and Pfam [55] databases. Only sequences that exhibited at least one characteristic amino acid sequence of the WD40 repeat were retained.

In addition to *R. simsii*, we selected 21 other plant species to investigate the evolutionary dynamics of the WD40 family across the plant kingdom, representing a range of lower to higher plants. The acquisition of WD40 protein sequences from several species followed the protocol described in "Investigation of WD40 TF Family in R. simsii and Other Plant Species" section. The selected species include *Medicago truncatula*, *Populus trichocarpa*, *Brassica rapa*, *Theobroma cacao*, *Vitis vinifera*, *Solanum lycopersicum*, *Solanum melongena*, *Camellia sinensis*, *Sorghum bicolor*, *Azolla filiculoides*, *Salvinia cucullate*, *Selaginella moellendorffii*, *Physcomitrium patens*, *Chlamydomonas reinhardtii*, *Ostreococcus lucimarinus*, and *Cyanidioschyzon merolae*. Additionally, we retrieved WD40 protein sequences of the following species from previously published studies: *Cucumis sativus* [6], *Malus domestica* [12], *Arabidopsis thaliana* [6], *Oryza sativa* [5], and *Ginkgo biloba* [7]. The evolutionary relationships among these species were determined based on the PGDD database [56] and a previous study [57].

### Characterization and genome distribution of RsWD40s

The coding sequence (CDS) and protein sequence of the RsWD40 TFs were obtained from RPGD database. To analyze their physical characteristics, all RsWD40 sequences were submitted to EXPASY (https://web.expasy.org/protparam/) for calculating the number of amino acids, molecular weight (MW), theoretical isoelectric point (pl), and grand average of hydropathicity (GRAVY) [58]. The position annotation of the *RsWD40* genes was obtained from the RPGD database and visualized using TBtools software [59].

### Classification and phylogenetic analysis of RsWD40s

To categorize the WD40 TFs family, two methods were employed: domain composition analysis and phylogenetic analysis. For the domain composition analysis, the amino acid sequences of the RsWD40 proteins were searched against the SMART database [54] to identify their domain compositions. Based on their domain compositions, the RsWD40s were categorized into distinct subfamilies.

To construct the phylogenetic trees, the full-length sequences of RsWD40 proteins were used. First, the identified RsWD40 proteins were aligned using the T-Coffee software [60] with default parameters to ensure accurate sequence alignment. Then, an unrooted phylogenetic tree was constructed using the neighbor-joining method implemented by MEGA 7 [61]. The phylogenetic analysis employed the poisson model, pairwise deletion to handle gaps in the alignment, and 1,000 bootstrap replicates to assess the support for branching patterns. Subsequently, the resulting phylogenetic tree was visualized and enhanced using iTOL [62]. To assign RsWD40 proteins to specific clusters within the phylogenetic tree, a threshold of bootstrap values greater than 0.5 was applied, following the method described by Li et al. [6]. RsWD40 proteins exhibiting bootstrap values above this threshold were grouped together in the respective clusters.

### Gene duplication analysis

To analyze gene duplication events among *RsWD40* genes, we employed DupGen_finder [63]. This tool allows differentiation between various types of gene duplicates, including whole-genome duplication (WGD), tandem duplication, transposed duplication, proximal duplication, and dispersed duplication, using the default settings. The duplicated *RsWD40* gene pairs were visualized using TBtools software [59]. To assess the evolutionary dynamics of the duplicated gene pairs, *Ka* (non-synonymous substitution rate), *Ks* (synonymous substitution rate), and *Ka*/*Ks* values were calculated using KaKs_Calculator (version 2.0) [64]. These values provide insights into the selective pressures acting on duplicated genes. Furthermore, we examined the synteny of *WD40* genes between *R. simsii* and other plant species using MCScanX [65]. The results of the synteny analysis were presented visually using TBtools software [59], allowing for a comprehensive understanding of the conservation and evolutionary relationships of *WD40* genes across different plant species.

### Expression analysis of *RsWD40* genes

To investigate the expression patterns of *RsWD40* genes, RNA-seq data from *Rhododendron* flower samples of three varieties (*Rhododendron wardii* var. puralbum, *Rhododendron simsii* Planch, and *Rhododendron hybridum* Ker Gawl, Figure S1), with white, pink, and red colored flowers, respectively, were analyzed. The plant materials used in this study were provided by Professor Xiaojing Wang from Guizhou University and were grown under controlled conditions in a growth chamber. Flower samples were collected at the bud and full bloom stages. The transcript abundance of *RsWD40* genes was quantified using the Fragments per kilobase of exon model per million mapped reads (FPKM) method. The expression data for each gene were normalized by log2 (FPKM) and presented as heat maps using TBtools software [59]. Differentially expressed genes (DEGs) were identified by comparing the expression levels between the different *Rhododendron* varieties. Genes with an absolute |fold change|≥ 2 (log2 |fold change|≥ 1) and a *p*-value < 0.05 were considered as DEGs. To identify candidate genes associated with anthocyanin biosynthesis, a comparative strategy was employed. Specifically, the gene expression data from the same stages of floral development were used to compare colored and white flowers (WF versus RF, WF versus PF), resulting in two sets of DEGs that included both up- and down-regulated genes. The final set of DEGs was obtained by intersecting these two sets. The comparative strategy was applied separately for the bud and full bloom stages.

### Confirmation of RNA seq analysis by qRT-PCR

To validate the RNA-seq results, qRT-PCR was conducted to validate the expression levels of candidate *RsWD40* genes related to anthocyanin biosynthesis at the bud and full bloom stages. The primers for qRT-PCR were designed using Beacon Designer 8, and their specific primer sequences can be found in Table S1. To ensure the accuracy and reliability of our experimental results, we adopted a strategy involving the use of at least two reference genes for data normalization [66–68]. The selection of *RsGAPDH* (Rhsim12G0106200) and *RsEF1α* (Rhsim02G0008200) as internal reference genes was based on their consistent expression levels in the RNA-seq data from the flowers of three different *Rhododendron* varieties at both developmental stages. Additionally, these genes have been utilized in previous studies investigating gene expression in the *Rhododendron* species [69, 70]. The qRT-PCR was carried out using the StepOnePlus™ System (Applied Biosystems, Foster City, CA, USA). The cycling parameters for qRT-PCR were as follows: 95 °C for 30 s, 95 °C for 5 s (40 cycles), and 60 °C for 30 s. Each qRT-PCR test included three biological and three technical replicates. The qRT-PCR reaction mixture was prepared using the TB Green® Premix Ex Taq™ kit (TaKaRa, Dalian, China). The data analysis was performed using the 2^−ΔΔCt^ method [71].

### GO enrichment analysis of candidate *RsWD40* genes

GOATOOLS (http://github.com/tanghaibao/GOatools) was used to perform gene annotation, and Fisher's exact test was employed to analyze the biological function enrichment of the candidate *RsWD40* genes. To reduce the likelihood of false positives, we applied the Bonferroni multiple testing correction, with a significance threshold set at a corrected p-value of less than 0.05. The results were visualized using a bubble dot diagram, which was generated on the website http://www.bioinformatics.com.cn.

## Result

### Identifcation and chromosomal locations of the RsWD40s

**Fig. 1:**
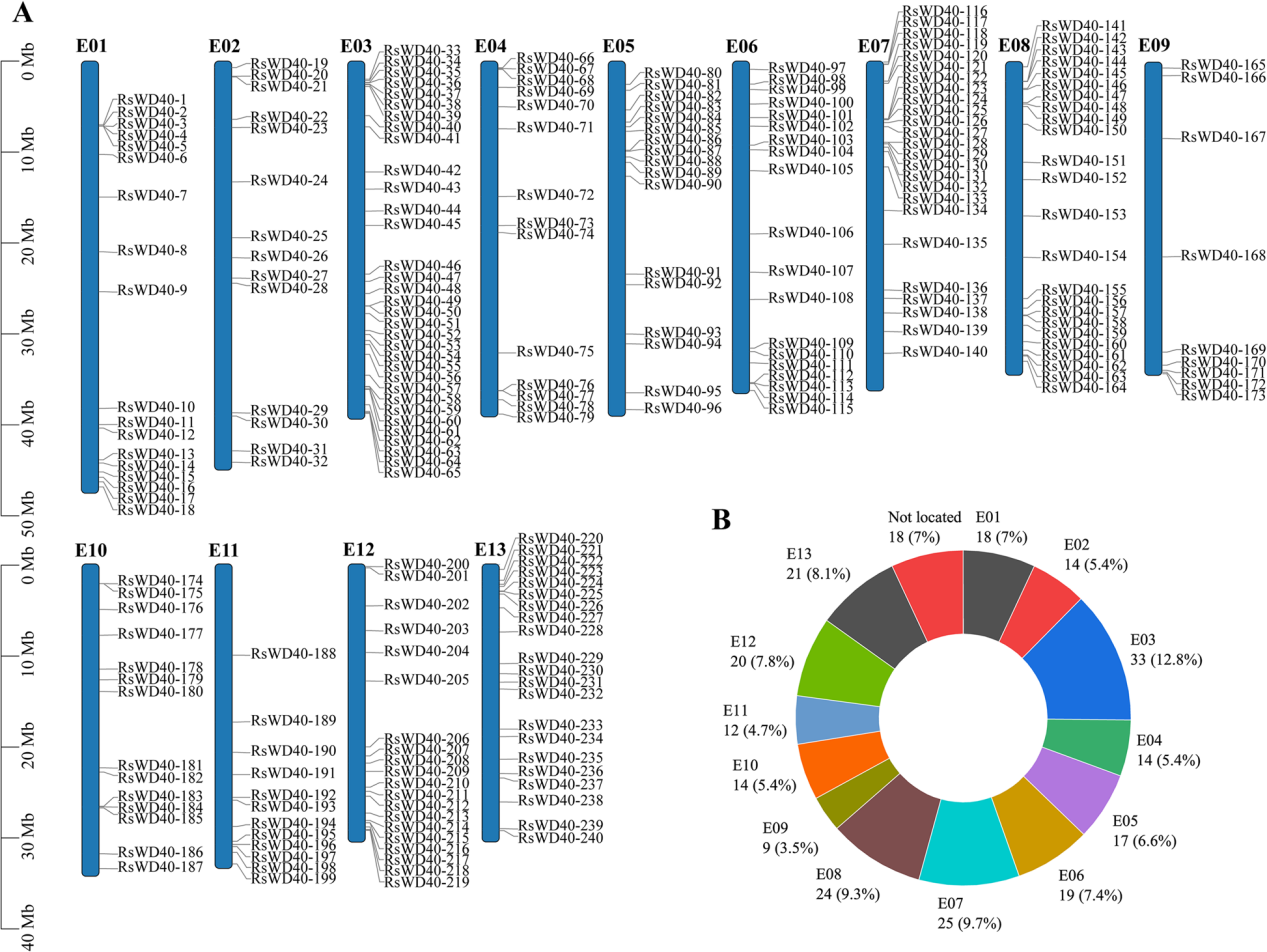
Chromosomal locations of the *RsWD40s*. The relative length of chromosomes serves as a measure of its size. **A** The distribution *RsWD40* gene on 13 chromosomes. **B** Numbers of *RsWD40* genes on each *R. simsii* chromosome

A total of 258 *RsWD40* genes were identified and renamed as *RsWD40-1* to *RsWD40-258* based on their chromosomal positions on Chr1-13 and the order of scaffolds (Fig. 1, Table S2). These genes were analyzed for their protein characteristics, including length, isoelectric point (PI), molecular weight (MW), and grand average of hydropathicity (GRAVY). The amino acid number of the RsWD40 proteins ranged from 94 (RsWD40-151) to 3671 (RsWD40-226). The putative PIs of RsWD40 proteins ranged from 4.40 (RsD40-70) to 9.76 (RsWD40-63). The MW of RsWD40 proteins varied from 10.95 kDa (RsWD40-151) to 408.65 kDa (RsWD40-226). The vast majority of RsWD40 proteins (248 out of 258) had a GRAVY (grand average of hydropathicity) value lower than 0, indicating they were hydrophilic. As shown in Fig. 1, the distribution of 240 *RsWD40s* (with the remaining 18 not being located) across the 13 chromosomes varies, with the maximum 33 *RsWD40s* found on chromosome 3, and only 9 *RsWD40s* found on chromosome 9.

### Classification of RsWD40 TFs

The RsWD40 transcription factors (TFs) exhibit a diverse range of protein domains in addition to the WD40 repeats, as summarized in Table S1. Considering the presence of these additional domains, we classified the 258 RsWD40 TFs into 42 distinct subfamilies, as shown in Table S3. Subfamily 1 (S1) consisted of 162 RsWD40 TFs that exclusively contained WD40 repeats, while the remaining 96 RsWD40 TFs sharing common domains were assigned to Subfamilies 2–42 (Table S3).

**Fig. 2:**
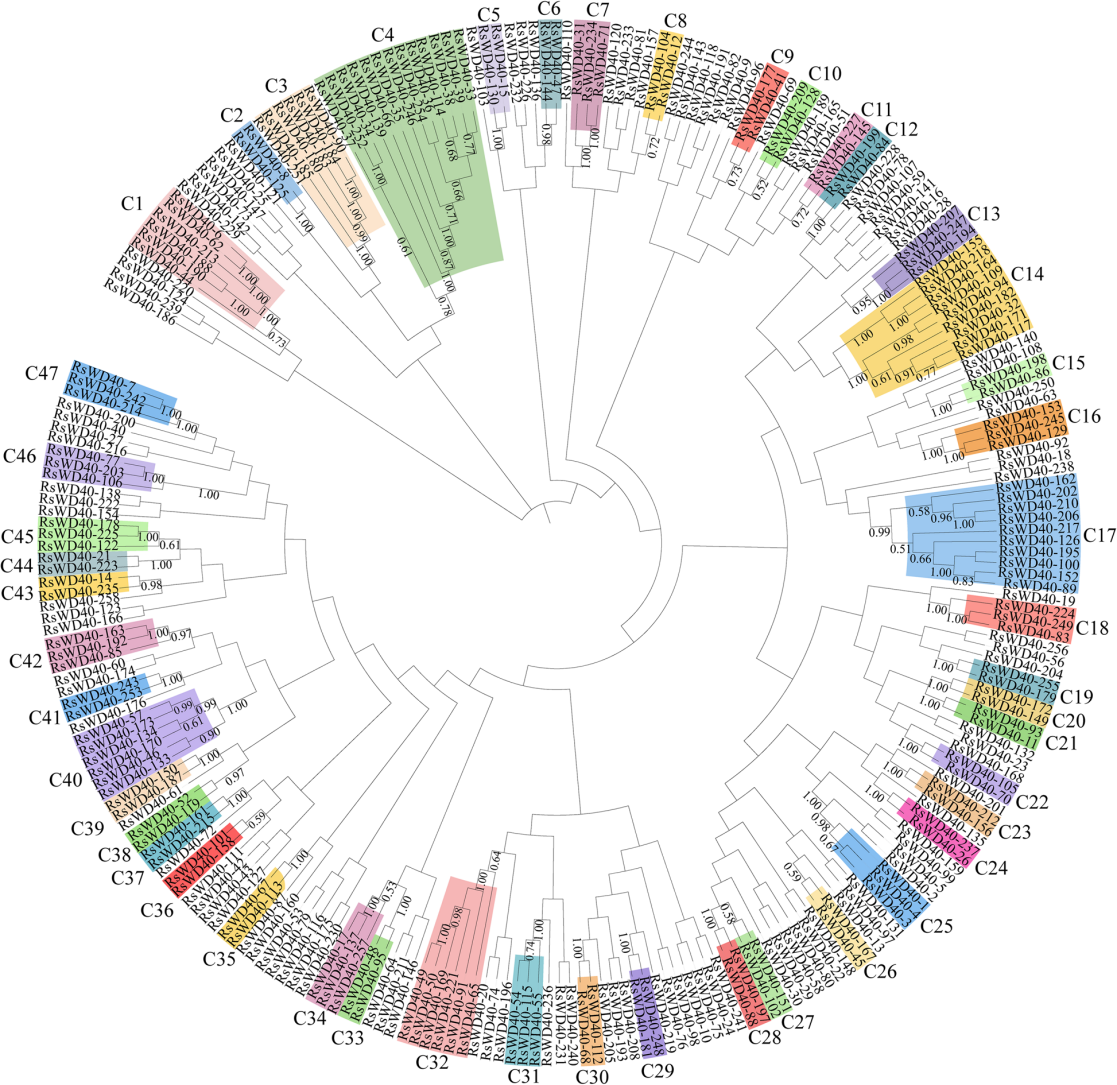
Phylogenetic analysis of WD40 proteins in *R. simsii*. The phylogenetic tree was constructed using the neighbor-joining method implemented by MEGA 7, based on the complete amino acid sequences of RsWD40 proteins. The tree shows 47 phylogenetic clusters (C1-C47) with high bootstrap values. Bootstrap values lower than 0.5 are not displayed in the phylogenetic tree

Due to variations in the number of WD40 repeats and the spacing of amino acids between them, aligning the WD40 repeats directly posed challenges. To overcome this, we employed the complete amino acid sequences of RsWD40 proteins to construct an unrooted phylogenetic tree using MEGA 7 software. Figure 2 depicts the identification of 47 clusters (C1–C47) among the 258 RsWD40 proteins, with a minimum bootstrap support of 0.5. Notably, out of the 258 RsWD40 proteins, 112 could not be definitively assigned to any specific cluster through the phylogenetic analysis.

### Comparative genomic analysis of *WD40* genes

According to our investigation, we have identified a total of 258, 286, 332, 348, 243, 255, 262, 262, 323, 234, 227, 205, 204, 305, 223, 125, and 78 *WD40* genes from *R. simsii*, *M. truncatula*, *P. trichocarpa*, *B. rapa*, *T. cacao*, *V. vinifera*, *S. lycopersicum*, *S. melongena*, *C. sinensis*, *S. bicolor*, *A. filiculoides*, *S. cucullate*, *S. moellendorffii*, *P. patens*, *C. reinhardtii*, *O. lucimarinus*, and *C. merolae*, respectively (Table S4). Furthermore, previous studies have suggested that *C. sativus*, *M. domestica*, *A. thaliana*, *O. sativa*, and *G. biloba* species possess 191, 346, 230, 200, and 167 *WD40* genes, respectively [5–7, 12].

**Fig. 3:**
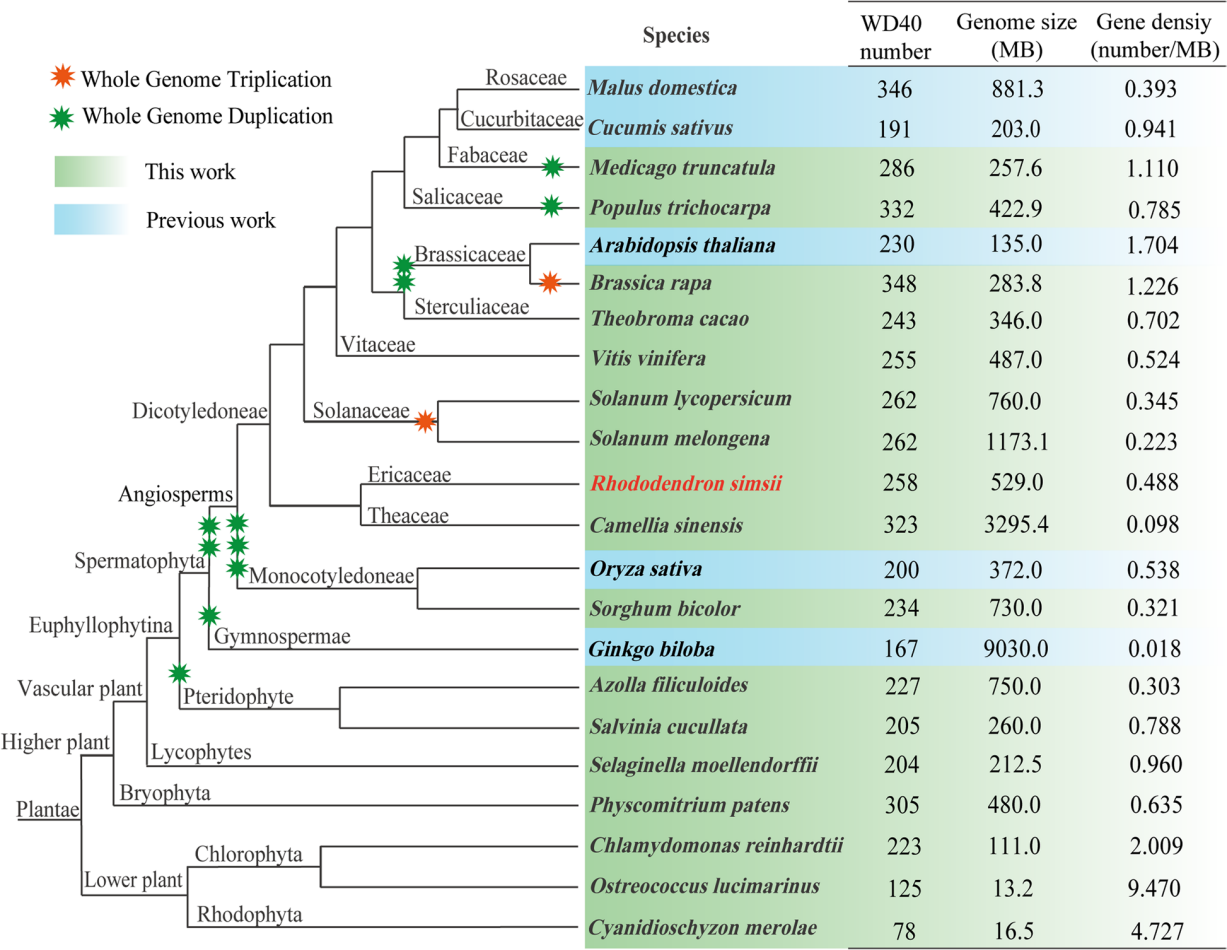
The evolutionary relationships of the 22 plant species and the specific information of the *WD40* gene family in each genome. The left side of the figure shows the evolutionary relationships of the species, the right side shows the number of *WD40* gene family members in each species. Green data indicate information described in this work, while blue data indicate information previously published

To explore the evolutionary patterns of the *WD40* gene family, we conducted a comparative genomic study of *WD40* genes in these 22 plant species. Figure 3 illustrates the evolutionary relationships among these species and the corresponding number of *WD40* genes in each genome. Our findings indicated that *WD40* genes are abundant in both lower and higher plants. Additionally, we calculated the gene density of *WD40* and observed that it was higher in lower plants compared to higher plants. In higher plants, the gene density of *WD40* decreased starting from *P. patens* and then remained relatively stable. Interestingly, *G. biloba* had the lowest *WD40* gene density among all species due to its enormous genome size.

### Gene duplication and *Ka*/*Ks* analysis

**Fig. 4:**
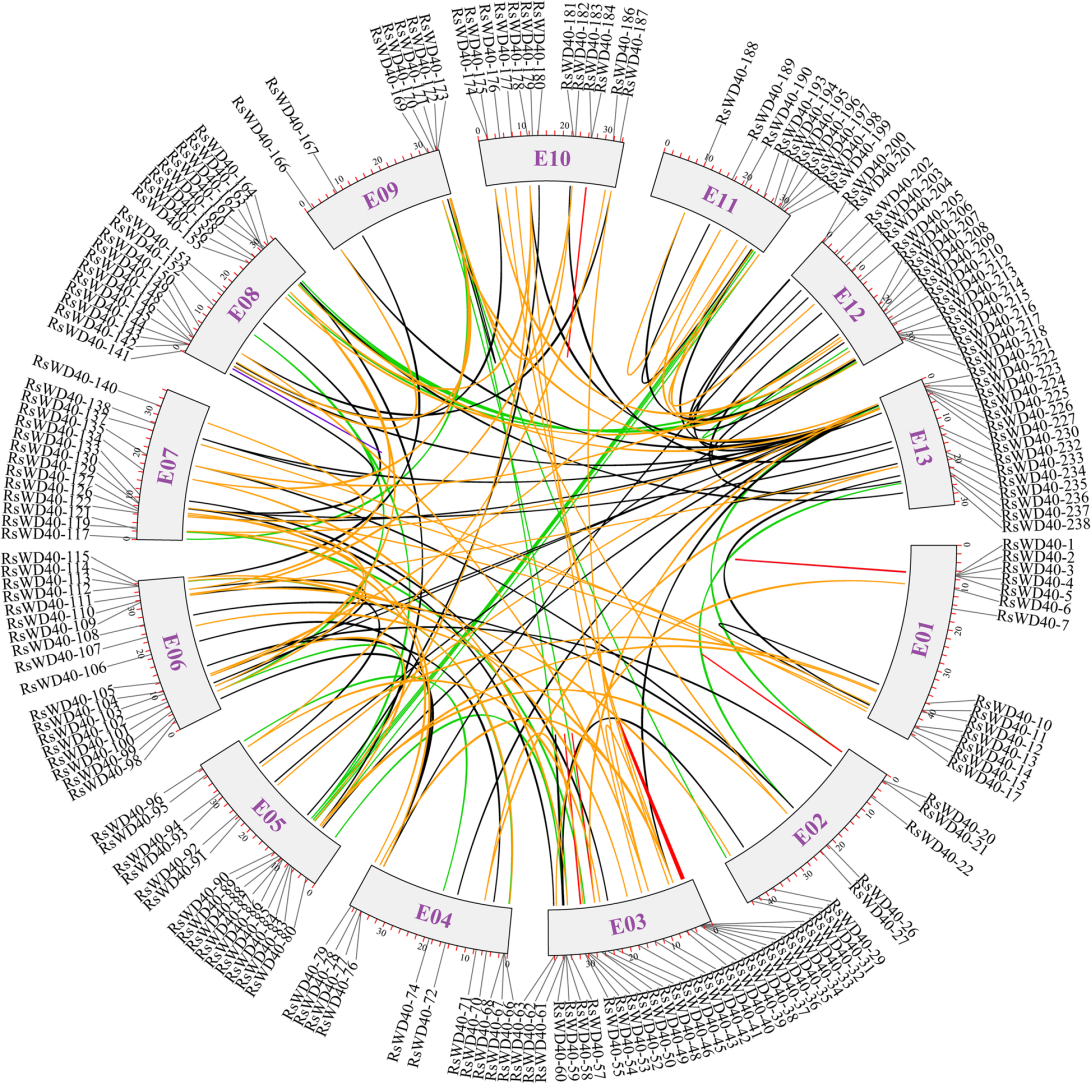
Genomic locations of *RsWD40s* and all duplication gene pairs in the *R. simsii* genome. Duplicated *RsWD40* gene pairs were indicated by the colored lines: WGD duplication pairs (green), tandem duplication pairs (purple), proximal duplication pairs (red), transposed duplication pairs (black), dispersed duplication pairs (orange). Color boxes with a number inside it represents chromosomes

Gene duplication events were classified into five categories, namely WGD, tandem, proximal, transposed, and dispersed duplications, using DupGen_finder. Among these duplication events, transposed duplication accounted for the highest proportion at 33.7%, followed by dispersed duplication (23.6%), WGD (19%), proximal duplication (6.2%), and tandem duplication (5%). Additionally, 32 RsWD40 genes (12.4%) were identified as singletons, indicating that they did not arise from duplication (Figure S2, Table S2). A total of 152 duplicate gene pairs were identified among the *RsWD40s* (Table S5, Fig. 4). We further analyzed the synonymous (*Ks*) and non-synonymous (*Ka*) mutations in these gene pairs (Table S5). The *Ka*/*Ks* ratio was used as an indicator of selection pressure during evolution. The analysis revealed that the majority of *RsWD40* gene pairs exhibited a *Ka*/*Ks* ratio less than 1, suggesting the influence of purifying selection. This indicates that these genes have undergone functional constraints and selective pressure to maintain their essential functions. Only one gene pair (*RsWD40-246*/*RsWD40-254*) had a *Ka*/*Ks* ratio greater than 1, suggesting positive selection for advantageous mutations in these genes.

**Fig. 5:**
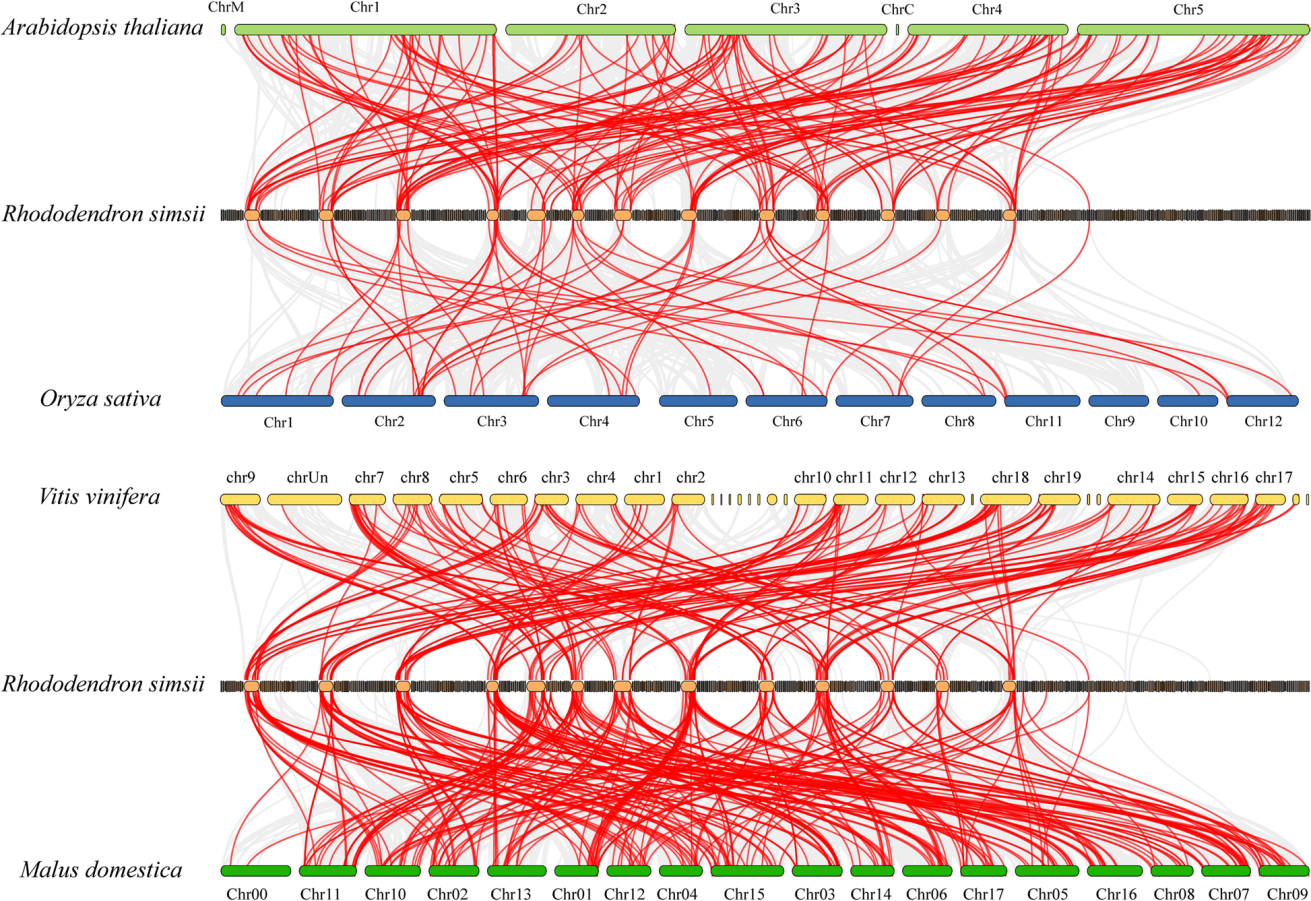
Comparative physical mapping displaying the orthologous relationships of *WD40* genes among *R. simsii* and other four species (*A. thaliana*, *O. sativa*, *V. vinifera* and *M. domestica*)

To investigate the potential evolutionary processes of the *WD40* gene family, we conducted a synteny analysis to examine orthologous relationships of *WD40* family genes between *R. simsii* and four other species (*A. thaliana*, *O. sativa*, *V. vinifera* and *M. domestica*) (Fig. 5). The analysis revealed a total of 164 pairs of orthologs between *R. simsii* and *A. thaliana*, 55 pairs of orthologs between *R. simsii* and *O. sativa*, 190 pairs of orthologs between *R. simsii* and *V. vinifera*, 306 pairs of orthologs between *R. simsii* and *M. domestica* (Table S6). These orthologous pairs of *RsWD40* genes and the corresponding *AtWD40*, *OsWD40*, *VvWD40* or *MdWD40* genes can be traced back to a common ancestor. The orthologous relationships varied, with some involving one *RsWD40* gene corresponding to one ortholog gene (e.g., *RsWD40-11*/AT3G45280 and *RsWD40-127*/LOC_Os01g72220), while others involved one *RsWD40* corresponding to multiple ortholog genes (e.g., *RsWD40-171*/AT5G24320/AT5G53500/AT5G54200 and *RsWD40-17*/LOC_Os02g50740/LOC_Os06g13140). These results suggest that duplication events have likely played a significant role in the evolution and functional diversification of the *WD40* family.

### Expression profiling of *RsWD40 *genes in three *Rhododendron* varieties

**Fig. 6:**
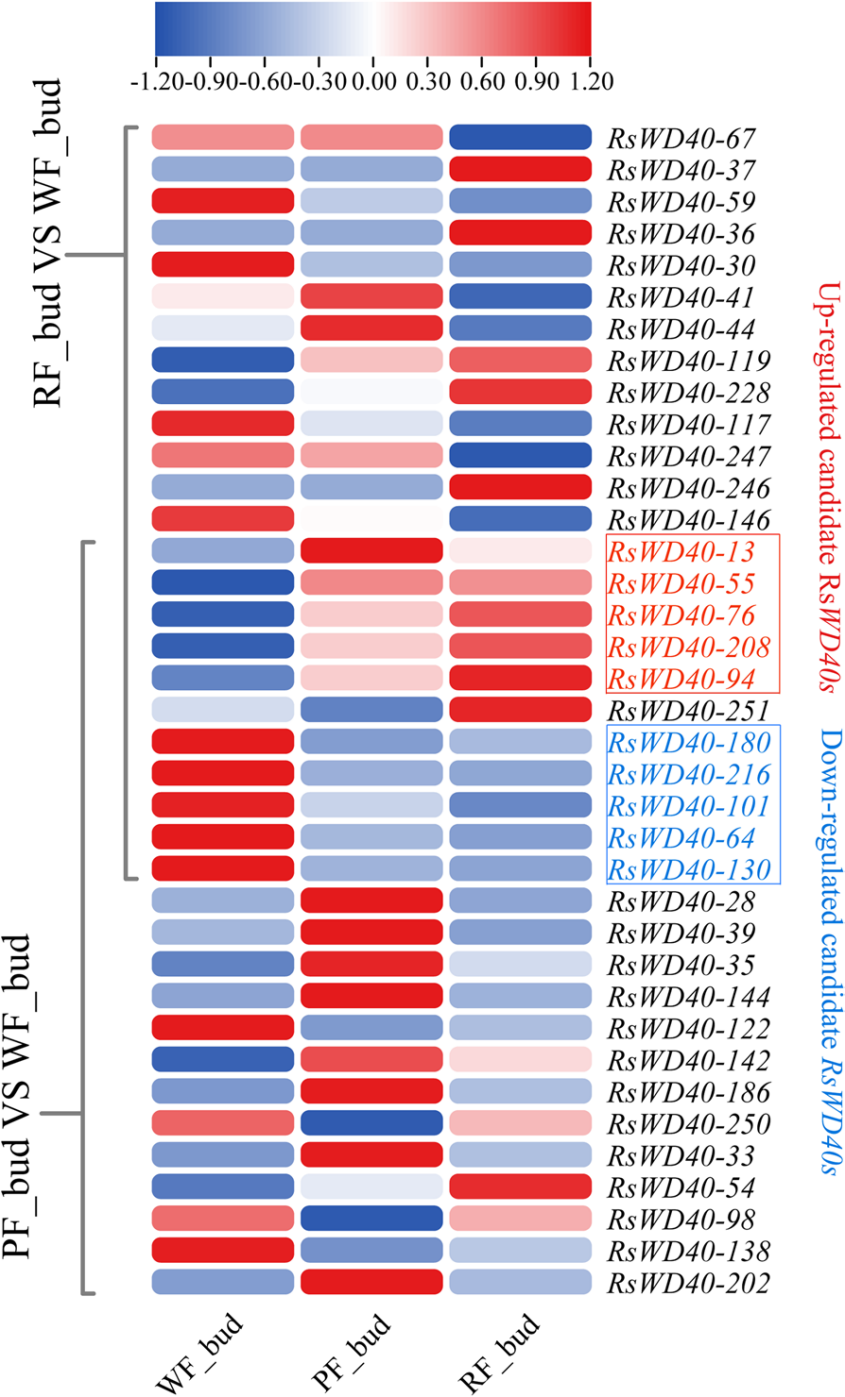
Identification of candidate *RsWD40s* involved in anthocyanin biosynthesis across three different flower colors (white, red, and pink) in *Rhododendron* varieties at the bud stage. The heatmap shows the relative expression values of *RsWD40s*, with WF indicating white-flowering variety, RF indicating red-flowering variety, and PF indicating pink-flowering variety

**Fig. 7:**
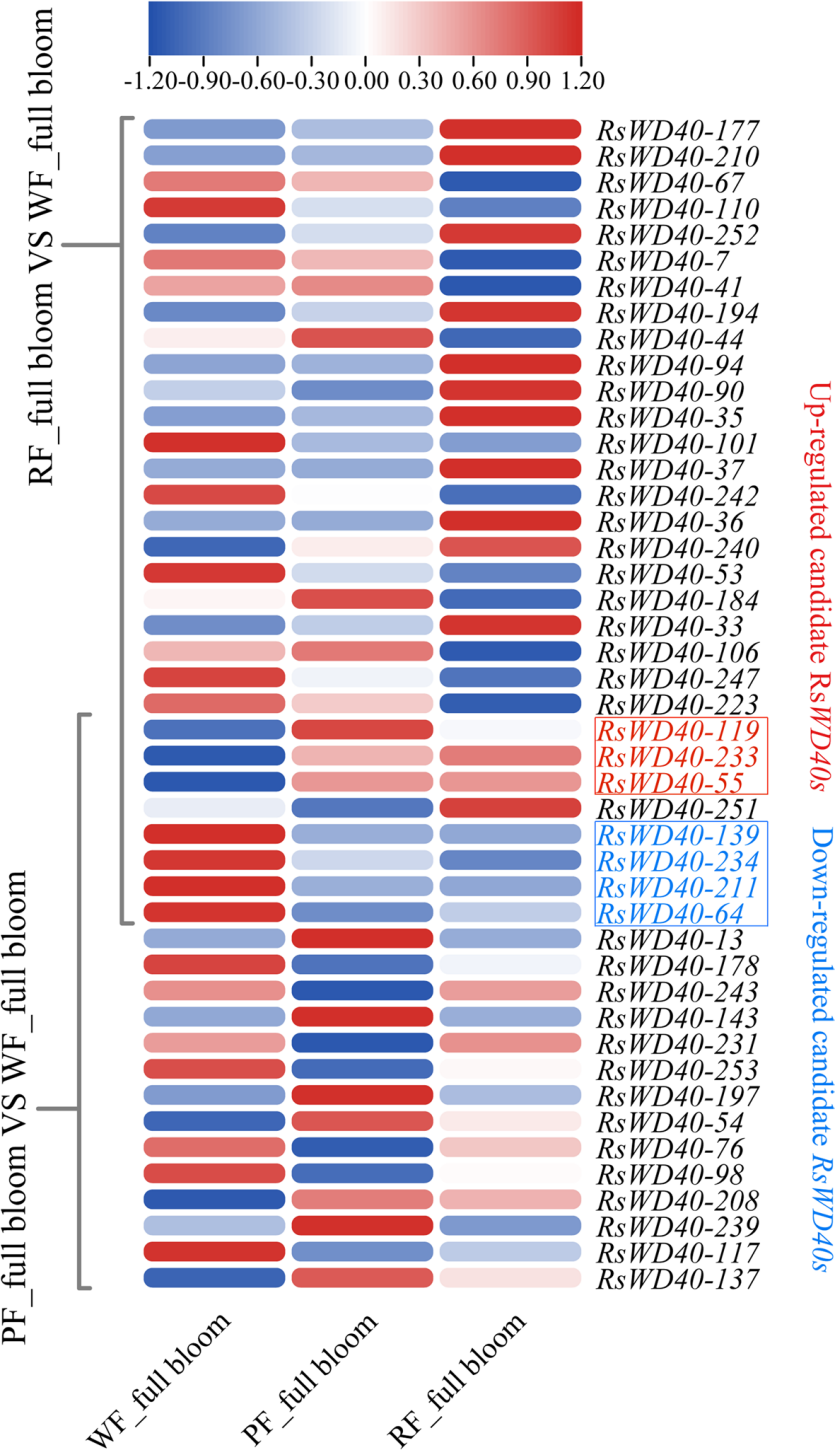
Identification of candidate *RsWD40s* involved in anthocyanin biosynthesis across three different flower colors (white, red, and pink) in *Rhododendron* varieties at the full bloom stage. The heatmap shows the relative expression values of *RsWD40s*, with WF indicating white-flowering variety, RF indicating red-flowering variety, and PF indicating pink-flowering variety

In this study, we utilized RNA-seq data to examine the expression profiles of *RsWD40* genes in three *Rhododendron* cultivars, aiming to identify *WD40* genes associated with anthocyanin biosynthesis in *R. simsii*. We identified differentially expressed genes (DEGs) with absolute |fold change|≥ 2 (log2 |fold change|≥ 1) and p-value < 0.05 between the two *Rhododendron* varieties. Using this approach, we identified 24 DEGs between red flower (RF) and white flower (WF) varieties, and 24 DEGs between pink flower (PF) and white flower (WF) varieties, at the bud stage. By combining these two sets of DEGs, we aimed to identify candidate RsWD40s involved in anthocyanin biosynthesis. Our analysis revealed the up-regulation of 5 DEGs (*RsWD40-13*, *55*, *76*, *94*, and *208*) in colored-flower (PF and RF) varieties. This suggests that these genes may positively regulate anthocyanin production at the bud stage. On the other hand, the down-regulation of 5 DEGs (*RsWD40-64*, *101*, *130*, *180*, and *216*) in colored-flower (PF and RF) varieties indicates their potential involvement in negatively regulating anthocyanin biosynthesis at the bud stage (Fig. 6, Table S7 and S8). In addition, we conducted a similar comparative analysis at the full bloom stage. We identified 3 candidate genes (*RsWD40-55*, *119*, and *233*) that may play a role in positively regulating anthocyanin production. Furthermore, we found 4 candidate genes (*RsWD40-64*, *139*, *211*, and *234*) that could potentially be involved in negatively regulating anthocyanin biosynthesis (Fig. 7, Tables S9 and S10).

### Validation of RNA-Seq-based gene expression

**Fig. 8:**
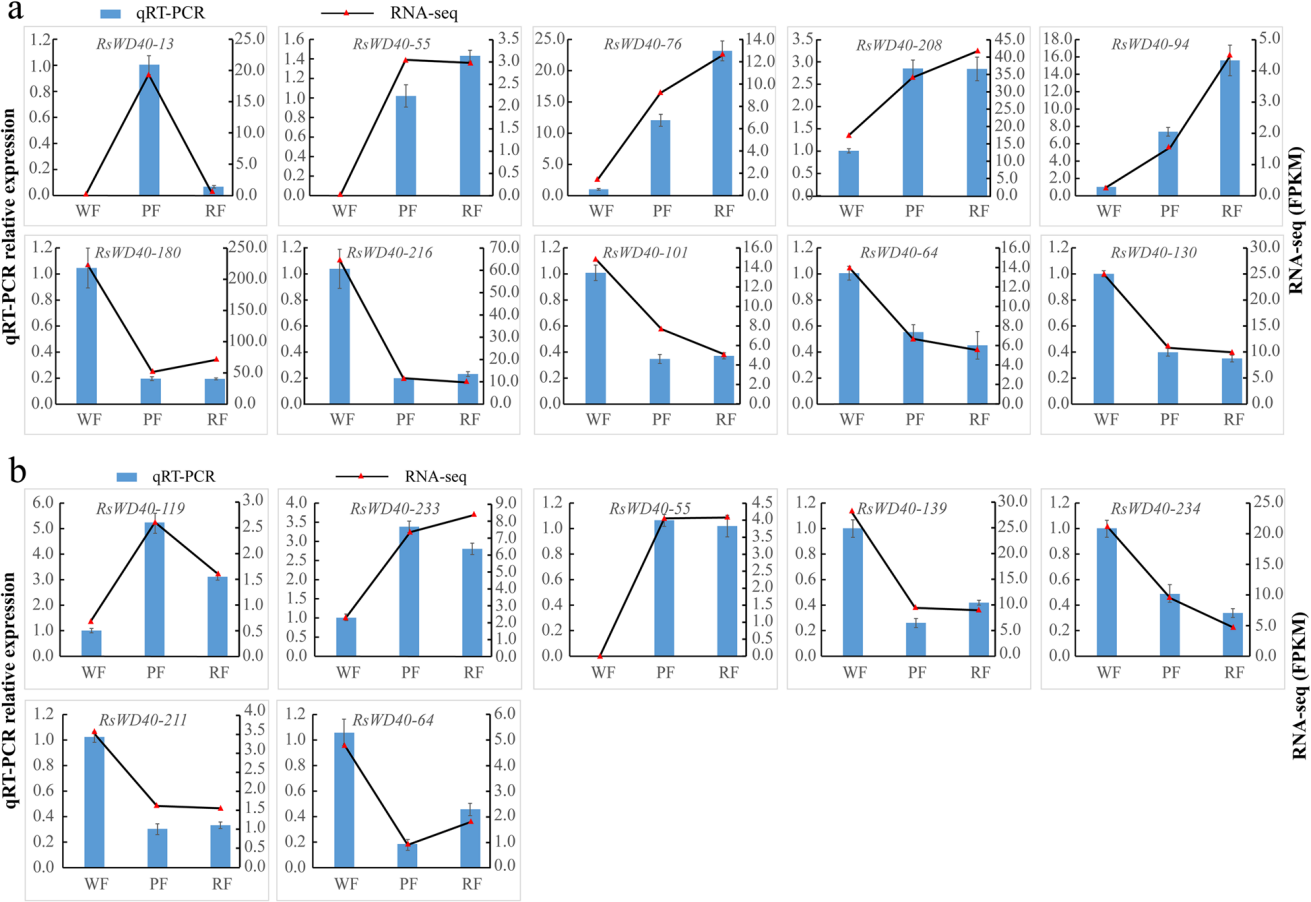
qRT-PCR validation of 15 candidate *RsWD40* genes at the bud stage (**a**) and full bloom stage (**b**). WF, PF, and RF represent white-flowering variety, pink-flowering variety, the red-flowering variety, respectively. Data represent the mean of three biological replicates ± standard error of the mean. Standard errors are shown as bars above the columns

To confirm the accuracy of the RNA-seq results, we selected and examined 15 candidate genes related to anthocyanin biosynthesis at the bud and full bloom stages using qRT-PCR. It is important to note that the consistency between qRT-PCR and RNA-seq results may vary for different genes. Previous studies, such as Everaert et al. [72], have demonstrated that qRT-PCR and RNA-seq results are not always perfectly aligned. Approximately 85% of genes exhibited consistent expression patterns between the two methods. In our study, despite the disparities in fold change values between the RNA-seq and qRT-PCR data, the overall expression trends remained consistent. For instance, based on the RNA-seq data, the expression of *RsWD40-76* was 8.0 and 5.9 times higher in red and pink flowers, respectively, compared to white flowers at the bud stage. However, in the qRT-PCR tests, the corresponding fold change values were 30.1 and 16.8. Additionally, some genes (*RsWD40-55*, *180*, *216*, and *101*) did not precisely follow the RNA-seq trend. Nevertheless, when comparing their expression levels between white flowers and colored flowers (including red and pink), both RNA-seq and qRT-PCR data demonstrated consistent results, despite potential differences in fold changes for red and pink flowers. In conclusion, the qRT-PCR results aligned well with the RNA-seq data in terms of the observed expression trends (Table S11, Fig. 8).

### GO enrichment analysis of candidate *RsWD40* genes

**Fig. 9:**
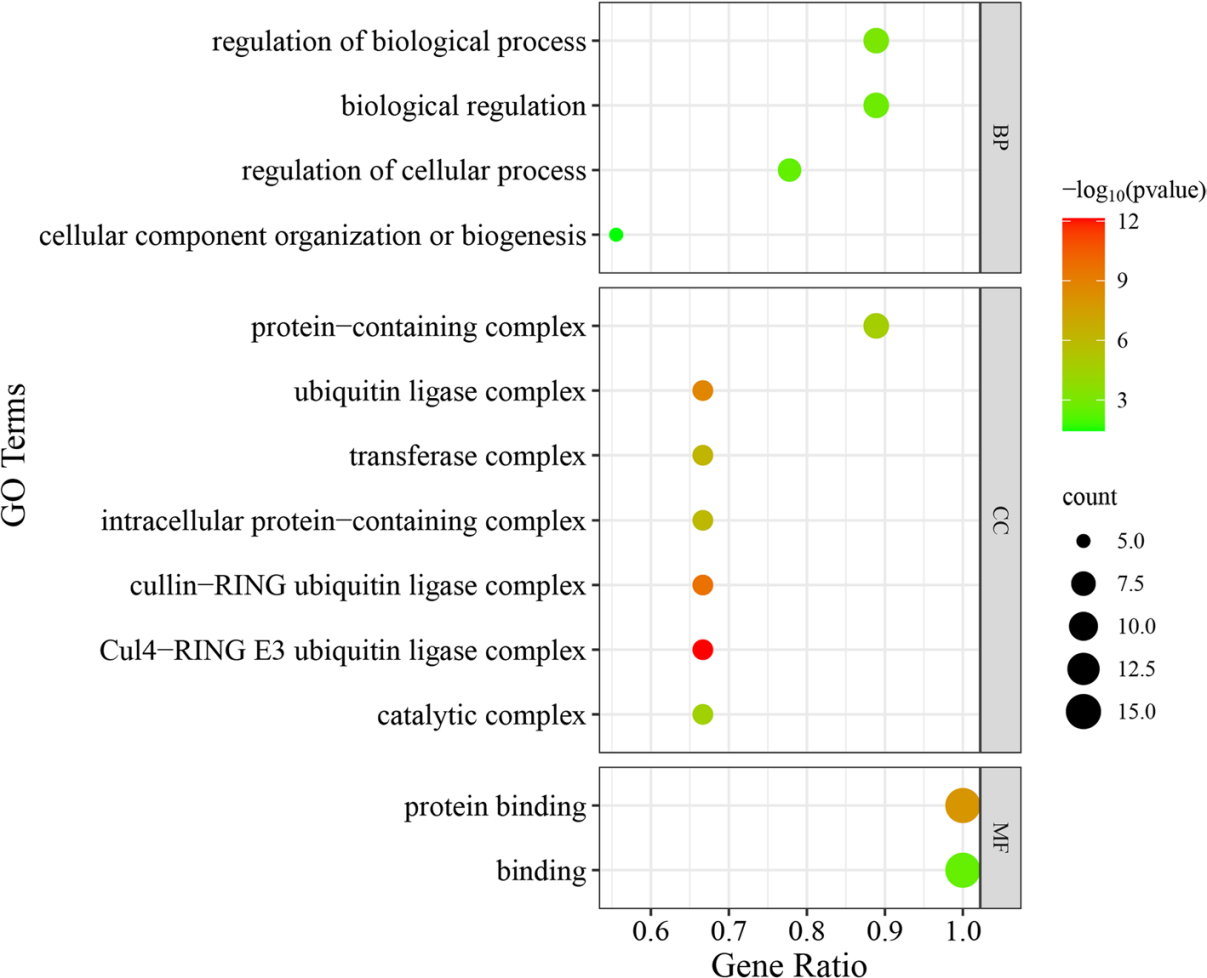
GO enrichment of the candidate *RsWD40* Genes. GO was performed with three main categories: molecular function (MF), cellular component (CC) and biological process (BP). GO terms with *P* value < 0.05 were identified as significant

In order to comprehensively understand the biological function of the candidate *RsWD40* genes, we conducted gene ontology (GO) annotation and enrichment analysis on the 15 candidate genes (*RsWD40-13*, *RsWD40-55*, *RsWD40-64*, *RsWD40-76*, *RsWD40-94*, *RsWD40-101*, *RsWD40-119*, *RsWD40-130*, *RsWD40-139*, *RsWD40-180*, *RsWD40-208*, *RsWD40-211*, *RsWD40-216*, *RsWD40-233*, and *RsWD40-234*) investigated in this study. Our analysis revealed significant enrichment for 13 GO terms, comprising of 2 molecular function terms, 7 cellular component terms, and 4 biological process terms (Fig. 9, Table S12). The two enriched molecular function terms were "binding" and "protein binding", indicating the involvement of these candidate genes in binding interactions. The four enriched biological process terms were "regulation of biological process", "biological regulation", "regulation of cellular process", and "cellular component organization or biogenesis", suggesting their roles in regulating various cellular processes. Notably, in the cellular component category, the most enriched GO terms were "Cul4-RING E3 ubiquitin ligase complex", "cullin-RING ubiquitin ligase complex", and "ubiquitin ligase complex". This finding suggests that ubiquitination process may play an important role in anthocyanin biosynthesis in *R. simsii*.

## Discussion

The grouping of gene families is crucial because genes within the same group often exhibit structural and functional similarities, making it easier to predict gene function. Previous studies have employed two approaches to group WD40 TFs families: by their domain composition or through phylogenetic analysis (see ‘Introduction’ section). The former approach involves grouping WD40 proteins into subfamilies based on their domain compositions. For instance, WD40 proteins from various species, including potato, wheat, apple, and *Ginkgo biloba*, have been classified into different subfamilies (ranging from 10 to 15) based on domain composition [7, 9, 12, 13]. However, some of these studies have classified WD40 proteins with different domain compositions into the same group, referred to as "other subfamilies", suggesting the possibility of further subfamily divisions. In our study, we conducted a more comprehensive grouping of RsWD40 proteins and identified 42 subfamilies. These results suggest that plant WD40 proteins contain a variety of domains in addition to the WD40 repeat. The second method involves grouping WD40 proteins based on phylogenetic analysis. Previous studies on WD40 proteins have reported low bootstrap values in phylogenetic trees for several species, including *Arabidopsis thaliana* [6], cucumber [6], *Setaria italica* [11], and potato [13], indicating poor support for many cluster assignments. Consequently, the grouping of WD40 proteins by phylogenetic analysis has exhibited significant variability among studies, even within the same species. For example, WD40 in *Arabidopsis* have been identified into 32, 14, 11, and 5 clusters in different studies [6, 13, 15, 73]. Here, we adopted a phylogenetic tree-based approach as previously described by Li et al. [6], which excluded genes with low support values to avoid incorrect assignments. As a result, we identified 47 distinct clusters for RsWD40s. It's important to note that the number of WD40 repeats in the WD40 protein can vary [74]. In our study, RsWD40 proteins displayed a range of 1 to 12 WD40 repeats. The variable number of WD40 repeats and the diversity of other domains within the WD40 family members likely contribute to reduced sequence conservation, which could account for the low bootstrap values observed in the phylogenetic tree.

To explore the evolution of the *WD40* gene family, we investigated the *WD40* gene family in *R. simsii* and 21 other species, including 18 higher plants and 3 lower plants. Our comparative genomic analysis revealed that single-celled *C. merolae*, *O. lucimarinus* and *C. reinhardtii* have 78, 125 and 223 *WD40* genes, respectively. The abundance of *WD40* genes present in lower plants suggests their involvement in fundamental cellular processes essential for plant survival. This may have aided lower plants in adapting to early extreme environments. Interestingly, the number of *WD40* genes in green algae (223) was found to be higher than that in higher plants such as rice (200), *C. sativus* (191), and *S. cucullata* (205). This suggests that no large-scale amplification of *WD40* genes occurred in higher plants and that the expansion of *WD40* gene family predates the divergence of green algae and higher plants. We also calculated the gene density of *WD40* genes in different plant species and observed that lower plants had a high gene density. Starting from *P. patens*, the gene density of *WD40* decreased and remained relatively stable in higher plants. *P. patens* is often used in evolutionary analyses as it allows for the reconstruction of genomic changes related to the conquest of land by comparing it with aquatic algae and vascular plants [75]. The reduced gene density of *WD40* in higher plants suggests that *WD40* gene loss occurred during evolution of these species. This indicates that *WD40* genes have undergone diversification over time and have acquired new functions in different plant lineages. For example, some *WD40* genes have been found to regulate flowering and pollen tube growth in higher plants [27, 76]. These functions may have emerged later in plant evolution, contributing to the diversification of plant lineages. Furthermore, we observed that *G. biloba* displayed an unusually low density of *WD40* genes compared to other species. This phenomenon can be attributed to the expansion of the *G. biloba* genome, which resulted in a significant increase in intron size primarily due to the insertion of long terminal repeats rather than recent whole-genome duplication events [57].

Gene duplication plays a crucial role in the rapid expansion and evolution of gene families [77]. Such duplication events can be classified into five categories: WGD, tandem duplication, proximal duplication, transposed duplication, and dispersed duplication [63]. The different patterns of gene duplication contribute differentially to the expansion of specific gene families in plant genomes [78, 79]. For example, some gene families, including *WRKY*, *bHLH*, and *bZIP*, are more likely to expand through WGD and tandem duplications [80–82], while others such as *NBS*-*LRR* and *ERF* expand primarily through transposed duplication [83–85]. Our research on the *WD40* gene family in *R. simsii* suggested that transposed duplication was the primary cause of its expansion, with 33.7% of the *RsWD40* genes duplicated and retained through transposed duplication. Duplication of genes can lead to diverse results such as pseudogenization (function loss), subfunctionalization, and neofunctionalization [86]. At both bud and full bloom stages, the gene pair *RsWD40-20*/*RsWD40-223*, resulting from WGD, exhibited different levels of expression. This suggests that duplicated genes may remain stable if they have different functional properties, which have been shaped by natural selection during the process of evolution. We also analyzed the rate of synonymous (*Ks*) to non-synonymous (*Ka*) mutation for all duplication gene pairs. Typically, genes are subject to three types of selection: purifying selection (*Ka*/*Ks* < 1), positive selection (*Ka*/*Ks* > 1), and neutral selection (*Ka*/*Ks* = 1) [87]. Our study observed that the *Ka*/*Ks* ratio of almost all *RsWD40* gene pairs was less than 1, indicating that these genes are mostly affected by purifying selection. Only one gene pair had a *Ka*/*Ks* ratio greater than 1, suggesting positive selection for advantageous mutations. Genomic comparison is an efficient means of transferring genomic knowledge of a well-studied taxonomic unit to a less studied one [88]. Our synteny analysis revealed 164 orthologous gene pairs between *R. simsii* and *A. thaliana*, 55 pairs with *O. sativa*, 190 pairs with *V. vinifera*, and 306 pairs with *M. domestica* (Table S6). This indicates that these gene pairs exhibit orthologous relationships, share common ancestors, have been conserved throughout evolution, and likely perform similar functions. The *WD40* genes identified in *A. thaliana, O. sativa, V. vinifera, and M. domestica* will serve as a reference for future studies of *RsWD40* genes.

Flower color is a crucial trait in plant breeding, and identifying genes associated with anthocyanin synthesis is essential for successful flower color breeding. The *WD40* gene *TTG1* in *A. thaliana* is well characterized for its involvement in anthocyanin synthesis, as discussed in the 'Introduction' section. However, *A. thaliana* lacks pigmentation, making it less suitable for studying anthocyanin synthesis. While some *TTG1* homologs have been studied in other species [44–48], a comprehensive genome-wide screening of *WD40* genes involved in anthocyanin synthesis in species with rich coloration has not been conducted. Therefore, in this study, we conducted a genome-wide screen for *WD40* genes involved in anthocyanin synthesis in *R. simsii* using RNA-seq data. The results of our study suggest that 10 candidate *RsWD40* genes may be involved in anthocyanin synthesis during the bud stage, and 7 candidate *RsWD40* genes may be involved in anthocyanin synthesis during the bloom stage. Interestingly, there was little overlap between these two groups of genes, with only *RsWD40-55* and *RsWD40-64* shared between them. This finding indicates that different *RsWD40* genes may have distinct roles in anthocyanin biosynthesis during different flowering stages. Previous studies have reported the involvement of the E3 ubiquitin ligase-mediated ubiquitin pathway in anthocyanin biosynthesis in various plants [89–94]. Our GO enrichment analysis revealed that terms related to the "E3 ubiquitin ligase complex" were the most enriched GO terms, and a total of 6 candidate genes (*RsWD40-234*, *RsWD40-180*, *RsWD40-130*, *RsWD40-101*, *RsWD40-208*, and *RsWD40-233*) were associated with these terms. These results suggest that the ubiquitination process may also play an important role in anthocyanin biosynthesis in *R. simsii*. In *R. simsii*, the homologous gene for *AtTTG1* (AT5G24520) is *RsWD40-172* (Rhsim09G0209900) (they are orthologous gene pair, Table S6). However, its expression did not significantly differ across all three floral colors during the budding and blooming stages. This suggests that *RsTTG1* may not be responsible for anthocyanin biosynthesis in *Rhododendron*, and other *RsWD40* genes may have taken on its function. Most of the up-regulated candidate *RsWD40* genes (7 of 8, Tables S9 and S11) identified in this study contained only WD40 repeat and had at least five WD40 repeats. These findings are consistent with the structural features of the AtTTG1 protein, implying a potential functional similarity between the candidate *RsWD40* genes and *AtTTG1*. However, further research is needed to confirm the potential involvement of the 15 candidate *RsWD40* genes related to anthocyanin biosynthesis.

## Conclusion

In conclusion, this study provides a comprehensive analysis of the WD40 transcription factor (TF) family in the *R. simsii* genome. We classified 258 WD40 TFs into 42 subfamilies and 47 clusters based on their domain compositions and phylogenetic analysis. The distribution of *RsWD40* genes was found to be uneven across the 13 chromosomes. Comparative genomic analysis suggests that the expansion of the *WD40* gene family occurred early in plant evolution, before the divergence of green algae and higher plants. Transposed duplication was identified as a major mechanism driving the expansion of the *RsWD40* gene family. Our analysis of selective pressure indicated that most duplication gene pairs underwent purifying selection. Through synteny analysis, we identified numerous orthologous gene pairs between *R. simsii* and other plant species, including *A. thaliana*, *O. sativa*, *V. vinifera*, and *M. domestica*. These findings highlight the conservation of certain genes and their potential functional similarities across different plant lineages. Furthermore, by utilizing RNA-seq data, we investigated the expression patterns of *RsWD40* genes and identified candidate genes potentially involved in anthocyanin biosynthesis during the bud and full bloom stages. GO enrichment analysis of these candidate genes revealed the potential involvement of the ubiquitination process in anthocyanin biosynthesis in *R. simsii*. Overall, this study enhances our understanding of the *RsWD40* family and provides valuable insights into the molecular mechanisms underlying anthocyanin biosynthesis in *Rhododendron* species.

### Supplementary Information

**Additional file 1: Figure S1.** The plant materials employed in this study, depicted from left to right, are *Rhododendron wardii *var. Puralbum, *Rhododendron simsii* Planch, and *Rhododendron hybridum* Ker Gawl.

**Additional file 2: Figure S2.** Proportion of genes originating from different replication events.

**Additional file 3: Table S1.** The primer sequences used for qRT-PCR. **Table S2.** The basic information of WD40 genes identified in Rhododendron simsii. **Table S3.** Grouping of RsWD40 proteins based on domain compositions. **Table S4.** The WD40 genes in typical species.  **Table S5.** Gene duplications of WD40 genes in R.simsii with outlier Ka/Ks values. **Table S6.** The orthologous relationships of the WD40 genes among Rhododendron simsii and other four species (Arabidopsis thaliana, Oryza sativa, Vitis vinifera and Malus domestica). **Table S7.** The expression of the RsWD40 at the bud stage. The gene expression levels were calculated by using reads per kilobase per million (RPKM) measure. **Table S8.** The differentially expressed genes at the bud stage. **Table S9.** The expression of the RsWD40 at the full bloom stage. The gene expression levels were calculated by using reads per kilobase per million (FPKM) measure. **Table S10.** The differentially expressed genes at the full bloom stage. **Table S11.** Relative expression of candidate RsWD40 genes at the bud and full bloom stages, as determined by qRT-PCR. **Table S12.** The gene ontology (GO) analysis of candidate RsWD40 genes.

## Data Availability

The RNA-seq data used in this study have been deposited in the National Center for Biotechnology Information (NCBI) Sequence Read Archive (SRA) database under the identifier PRJNA952839. The data are publicly available at http://www.ncbi.nlm.nih.gov/sra. The materials used in this research, including plant samples, are available upon request from the corresponding author.
