# Supplementary figures and images for: Genome-wide identification and bioinformatics analysis of the WD40 transcription factor family and candidate gene screening for anthocyanin biosynthesis in Rhododendron simsii
Source: BMC Genomics. 2023 Aug 26;24:488. doi: 10.1186/s12864-023-09604-x (PMC10463391; doi:10.1186/s12864-023-09604-x)

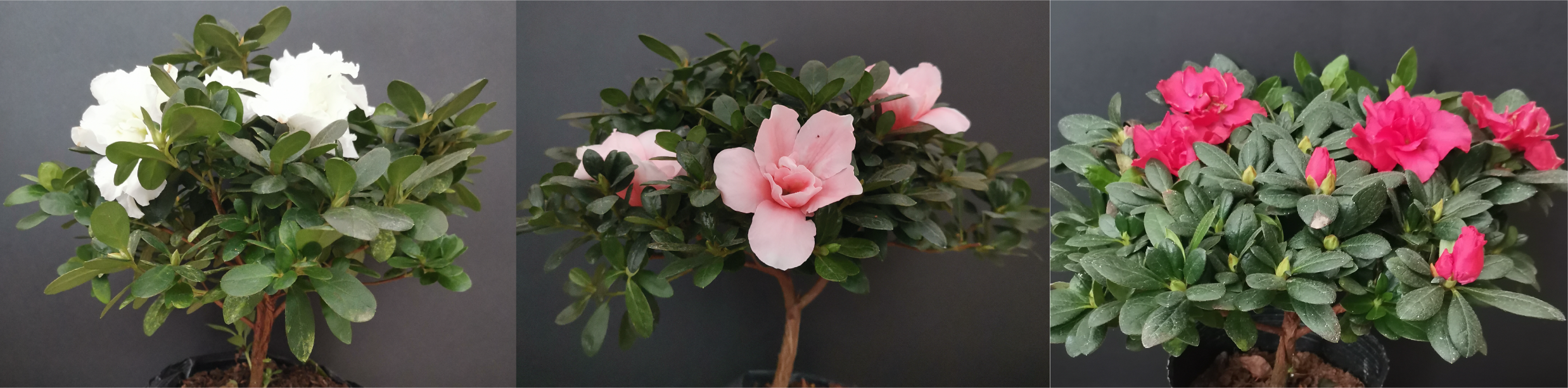

Supplement: Supplementary file 1 — Additional file 1: Figure S1. The plant materials employed in this study, depicted from left to right, are Rhododendron wardii var. Puralbum, Rhododendron simsii Planch, and Rhododendron hybridum Ker Gawl. [file 12864_2023_9604_MOESM1_ESM.tif]

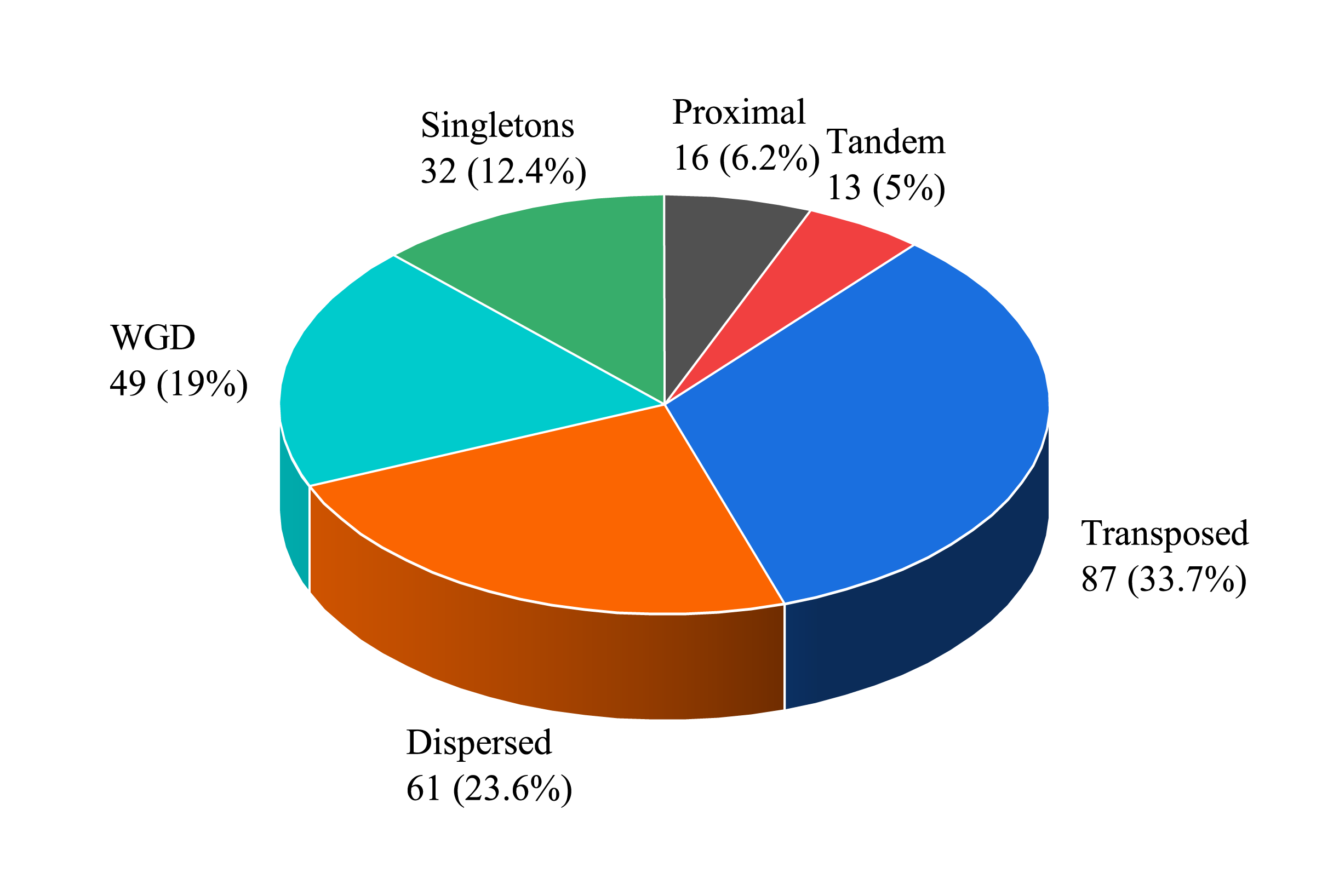

Supplement: Supplementary file 2 — Additional file 2: Figure S2. Proportion of genes originating from different replication events. [file 12864_2023_9604_MOESM2_ESM.tif]
